# Protein kinase C zeta promotes thyroid Cancer progression and represents a novel therapeutic target: evidence from specific atypical PKC inhibitor 2-acetyl-1,3-cyclopentanedione inhibitor studies

**DOI:** 10.3389/fmed.2025.1714626

**Published:** 2026-01-13

**Authors:** Jian Ding, Rongzhan Fu

**Affiliations:** 1 Department of Bun and Plastic Surgery, Shandong Provincial Third Hospital, Shandong University, Jinan, China; 2 Department of General Surgery, Shandong Provincial Qianfoshan Hospital, Shandong University, Jinan, China

**Keywords:** protein kinase C zeta, thyroid cancer, epithelial-mesenchymal transition, ACPD, therapeutic target

## Abstract

**Background:**

Poorly differentiated and anaplastic thyroid carcinomas represent aggressive malignancies with limited therapeutic options and poor prognosis. Protein kinase C zeta (PKCζ), an atypical PKC isozyme, has emerged as a critical regulator in various cancers, but its role in thyroid cancer progression remains largely unexplored. This study investigated PKCζ expression patterns in thyroid cancer and evaluated its therapeutic potential using the specific atypical PKC inhibitor 2-acetyl-1,3-cyclopentanedione (ACPD).

**Methods:**

PKCζ expression and phosphorylation were analyzed in thyroid tissue samples from 20 patients and multiple thyroid cancer cell lines using Western blot analysis. Functional studies employed PKCζ overexpression, knockdown, and ACPD treatment in BCPAP (papillary) and 8505C (poorly differentiated) cell lines. Cell proliferation, colony formation, migration, invasion, and epithelial-mesenchymal transition (EMT) markers were assessed. Therapeutic efficacy was evaluated in xenograft mouse models with ACPD treatment.

**Results:**

PKCζ expression and phosphorylation progressively increased from normal thyroid tissue through papillary, poorly differentiated, to anaplastic thyroid carcinomas. ACPD treatment (5 μM) significantly suppressed malignant phenotypes in 8505C cells, including reduced proliferation, colony formation, migration, and invasion, while reversing EMT marker expression. PKCζ knockdown reproduced these anti-tumorigenic effects, confirming specificity. PKCζ overexpression in BCPAP cells enhanced malignant behaviors, which were effectively counteracted by ACPD treatment. *In vivo* studies demonstrated that ACPD treatment significantly reduced tumor growth in both cell line-derived xenograft models.

**Conclusion:**

PKCζ activation correlates with thyroid cancer aggressiveness and drives malignant progression through EMT regulation. ACPD effectively targets PKCζ-mediated oncogenic pathways, suggesting PKCζ inhibition as a promising therapeutic strategy for aggressive thyroid cancers.

## Introduction

Thyroid cancer represents a significant and evolving global health challenge, with an estimated 44,020 new cases expected in the United States in 2025 (1). While the incidence rate has declined by about 2% each year since 2014 due to stricter diagnostic criteria, thyroid cancer remains the 13th most common cancer diagnosis overall and the 6th most common among women (2, 3). The disease encompasses distinct pathological subtypes with markedly different clinical outcomes: papillary thyroid carcinoma (PTC), follicular thyroid carcinoma (FTC), poorly differentiated thyroid carcinoma (PDTC), and anaplastic thyroid carcinoma (ATC) (4). While well-differentiated thyroid cancers generally have favorable prognoses, PDTC and ATC represent therapeutic challenges with limited treatment options and poor survival rates (5, 6). The 5-year survival rate for ATC remains below 10%, highlighting the urgent need for novel therapeutic targets and treatment strategies for these aggressive forms of thyroid cancer (7).

The protein kinase C (PKC) family comprises a critical group of serine/threonine kinases that serve as central mediators in cellular signal transduction pathways (8, 9). The PKC family consists of at least 12 isozymes classified into three major groups: conventional PKC isozymes (*α*, βI, βII, and *γ*) that require both calcium and diacylglycerol (DAG) for activation, novel PKC isozymes (*δ*, *ε*, *η*, and *θ*) that are calcium-independent but DAG-dependent, and atypical PKC isozymes (*ζ*, *ι*/*λ*) that are independent of both calcium and DAG (10). These kinases regulate diverse cellular processes including proliferation, differentiation, apoptosis, migration, and survival. PKC isozymes have been recognized as important players in carcinogenesis, with altered expression and/or dysregulation observed in multiple cancer types (11–13). The involvement of PKC in oncogenic processes has made this family an attractive target for cancer therapeutic development, with ongoing efforts to understand the specific roles of individual PKC isozymes in different malignancies.

Recent research has begun to elucidate the specific contributions of PKC isozymes to thyroid cancer progression (14). Early studies identified altered PKC activity in thyroid cancer, but recent investigations have provided more detailed insights into the roles of individual PKC subtypes (15, 16). A notable 2024 study by Campos Haedo and colleagues demonstrated that PKCα is overexpressed in thyroid carcinoma cell lines and mediates thyroid hormone-induced proliferation through the integrin αvβ3 receptor (17). Their findings revealed that PKCα-depleted cells significantly reduced thyroid hormone-induced proliferation through AKT and ERK activation pathways. While these studies have advanced our understanding of conventional PKC isozymes, the roles of different PKC subtypes in thyroid cancer appear to be highly context-dependent. Activation of PKCα and *β* isozymes have often been linked to malignant phenotype while PKCδ is thought to mediate anti-cancer effects, suggesting that individual PKC isozymes may have distinct and sometimes opposing functions in thyroid carcinogenesis (18). However, the specific contributions of atypical PKC isozymes, particularly PKCζ, to thyroid cancer progression remain largely unexplored.

PKCζ represents a unique member of the atypical PKC subfamily with distinctive biochemical and functional properties that differentiate it from conventional and novel PKC isozymes. Unlike conventional PKCs, atypical PKCs lack functional binding sites for DAG as well as calcium and are therefore independent of both for activation. This independence from classical second messengers allows PKCζ to be regulated through alternative mechanisms, including protein–protein interactions and distinct upstream signaling pathways (19, 20). Recent research has revealed that atypical PKC isozymes, including PKCζ, have pleiotropic context-dependent functions that can translate into either tumor-promoter or tumor-suppressive functions (11, 21, 22). In other cancer types, PKCζ has been implicated in critical processes such as epithelial-mesenchymal transition (EMT), cell polarity maintenance, and survival signaling. Studies have shown that PKCζ forms an apico-basal polarity complex with Partitioning Defective (Pard) 3 and Pard6 to regulate normal epithelial cell polarization, and dissociation of this complex is essential for EMT and tumor progression (23). Additionally, the availability of specific atypical PKC inhibitors such as 2-acetyl-1,3-cyclopentanedione (ACPD), which targets both PKCζ and PKCι, provides valuable pharmacological tools for investigating the therapeutic potential of targeting these kinases in cancer treatment (24).

The primary objective of this study was to investigate the role of PKCζ in thyroid cancer progression and evaluate its potential as a therapeutic target. We hypothesized that PKCζ serves as a key regulator of thyroid cancer malignancy, particularly through the modulation of EMT and associated malignant phenotypes. To test this hypothesis, we employed a comprehensive experimental approach combining both *in vitro* and *in vivo* methodologies, utilizing thyroid cancer cell lines representing different differentiation states, patient tissue samples, and xenograft mouse models. We investigated PKCζ expression patterns across thyroid cancer subtypes, examined the effects of PKCζ modulation on critical cellular processes including proliferation, migration, invasion, and EMT, and evaluated the therapeutic efficacy of the atypical PKC inhibitor ACPD. The findings from this investigation are expected to provide important insights into the molecular mechanisms underlying thyroid cancer progression and potentially identify PKCζ as a novel therapeutic target for aggressive thyroid cancers, which currently have limited treatment options and poor clinical outcomes.

## Materials and methods

### Ethics statement

This study was approved by the Ethics Committee of The Shandong Provincial Third Hospital (Jinan, China). Written informed consent was obtained from all patients before sample collection. All human experiments were performed in compliance with the Declaration of Helsinki. All animal experiments were performed in accordance with the guidelines approved by the Animal Care and Use Committee of The Shandong Provincial Third Hospital (Approval No. 202–230).

#### Patient samples and cell lines

Thyroid tissue samples were collected from 20 patients undergoing thyroidectomy at The Shandong Provincial Third Hospital between 2022 and 2024, including 5 normal thyroid tissues, 5 papillary thyroid carcinomas (PTC), 5 poorly differentiated thyroid carcinomas (PDTC), and 5 anaplastic thyroid carcinomas (ATC). Samples were immediately snap-frozen in liquid nitrogen and stored at −80 °C until use.

Human thyroid cancer cell lines 8505C, 8305C, SW579, and BCPAP, as well as the normal thyroid cell line Nthy-ori3-1, were obtained from the American Type Culture Collection (ATCC, Manassas, VA, USA). Cells were cultured in RPMI-1640 medium (Gibco, Grand Island, NY, USA) supplemented with 10% fetal bovine serum (FBS; Gibco) and 1% penicillin–streptomycin (Invitrogen, Carlsbad, CA, USA) at 37 °C in a humidified atmosphere with 5% CO_2_.

#### Reagents and antibodies

ACPD (2-acetyl-1,3-cyclopentanedione) was purchased from Sigma-Aldrich (St. Louis, MO, USA) and dissolved in DMSO at a stock concentration of 10 mM. For cell culture experiments, ACPD was used at a final concentration of 5 μM with fresh medium containing ACPD replaced every 24 h. Primary antibodies used were: anti-PKCζ (#9372, Cell Signaling Technology, Danvers, MA, USA), anti-phospho-PKCζ (pT560) (#ab62372, Abcam, Cambridge, UK), anti-PCNA (#13110, Cell Signaling Technology), anti-E-Cadherin (#3195, Cell Signaling Technology), anti-N-Cadherin (#13116, Cell Signaling Technology), anti-Vimentin (#5741, Cell Signaling Technology), anti-Snail (#3879, Cell Signaling Technology), and anti-*β*-actin (#4970, Cell Signaling Technology). HRP-conjugated secondary antibodies were from Jackson ImmunoResearch (West Grove, PA, USA).

#### Plasmid construction and transfection

The shRNA targeting PKCζ (sh-PKCζ), scrambled control shRNA (sh-Scr), PKCζ overexpression plasmid (PKCζ-OE), and empty vector control were purchased from Shanghai IBSBio (Shanghai, China). Cells were transfected using Lipofectamine 3000 (Invitrogen) according to the manufacturer’s instructions. Stable cell lines were selected using puromycin (2 μg/mL; Sigma-Aldrich) for 2 weeks.

#### Western blot analysis

Cells and tissues were lysed in RIPA buffer (Beyotime, Shanghai, China) supplemented with protease and phosphatase inhibitor cocktails (Roche, Basel, Switzerland). Protein concentration was determined using the BCA Protein Assay Kit (Pierce, Rockford, IL, USA). Equal amounts of protein (30 μg) were separated by 10% SDS-PAGE and transferred to PVDF membranes (Millipore, Billerica, MA, USA). Membranes were blocked with 5% non-fat milk for 1 h at room temperature and incubated with primary antibodies overnight at 4 °C. After washing, membranes were incubated with HRP-conjugated secondary antibodies for 1 h at room temperature. Protein bands were visualized using ECL reagent (Millipore) and quantified using ImageJ software (NIH, Bethesda, MD, USA).

#### Cell proliferation assay

Cell proliferation was assessed using the Cell Counting Kit-8 (CCK-8; Dojindo, Kumamoto, Japan). Cells (3 × 10^3^ cells/well) were seeded in 96-well plates and treated with vehicle control, DMSO, or ACPD (5 μM). Medium containing the respective treatments was refreshed every 24 h. At specified time points (0, 1, 2, and 3 days), 10 μL of CCK-8 solution was added to each well and incubated for 2 h at 37 °C. Absorbance was measured at 450 nm using a microplate reader (BioTek, Winooski, VT, USA).

#### Colony formation assay

Cells (500 cells/well) were seeded in 6-well plates and treated with vehicle control, DMSO, or ACPD (5 μM). Medium containing treatments was replaced every 24 h for the first 3 days, then every 3 days thereafter. After 14 days, colonies were fixed with 4% paraformaldehyde for 15 min and stained with 0.1% crystal violet (Sigma-Aldrich) for 30 min. Colonies containing more than 50 cells were counted under a microscope.

#### Transwell migration and invasion assays

Cell migration and invasion were assessed using Transwell chambers (8 μm pore size; Corning, NY, USA). For invasion assays, chambers were pre-coated with Matrigel (BD Biosciences, San Jose, CA, USA). Cells (5 × 10^4^) pre-treated with vehicle control, DMSO, or ACPD (5 μM) for 24 h were seeded in the upper chamber in serum-free medium containing the respective treatments, while medium containing 10% FBS was added to the lower chamber as a chemoattractant. After 24 h incubation at 37 °C, cells on the upper surface were removed with cotton swabs. Migrated or invaded cells were fixed with 4% paraformaldehyde, stained with 0.1% crystal violet, and counted in five random fields under a microscope (Olympus, Tokyo, Japan).

#### Wound healing assay

Cells were seeded in 6-well plates and grown to 90% confluence. A straight scratch was made using a 200 μL pipette tip. After washing with PBS to remove detached cells, fresh medium containing vehicle control, DMSO, or ACPD (5 μM) was added. Medium was refreshed every 24 h. Images were captured at 0 and 72 h using an inverted microscope (Leica, Wetzlar, Germany). The wound area was quantified using ImageJ software.

#### Xenograft tumor model

Male BALB/c nude mice (6–8 weeks old) were purchased from Charles River Laboratories (Beijing, China) and housed under specific pathogen-free conditions. For tumor growth experiments, 1 × 10^7^ cells suspended in 100 μL PBS were subcutaneously injected into the right flank of mice (*n* = 5 per group). For ACPD treatment groups, mice were intraperitoneally injected with ACPD (10 mg/kg/day) or vehicle control daily starting from day 7 post-injection. Tumor volume was measured every 7 days using calipers and calculated as: volume = (length×width^2^)/2. At the experimental endpoint (day 28), mice were euthanized with an intraperitoneal overdose of sodium pentobarbital (150 mg/kg), and tumors were excised and weighed.

### Statistical analysis

All experiments were performed in triplicate and repeated at least three times independently. Data are presented as mean ± standard error of the mean (SEM). Statistical analyses were performed using GraphPad Prism 9.0 (GraphPad Software, San Diego, CA, USA). Comparisons between two groups were analyzed using unpaired Student’s t-test. Multiple group comparisons were performed using one-way ANOVA followed by Tukey’s post-hoc test. Tumor growth curves were analyzed using two-way repeated measures ANOVA. *p* < 0.05 was considered statistically significant (*p < 0.05, **p < 0.01, ***p < 0.001).

## Results

### PKCζ expression and phosphorylation are progressively elevated in thyroid cancer

**Figure 1:**
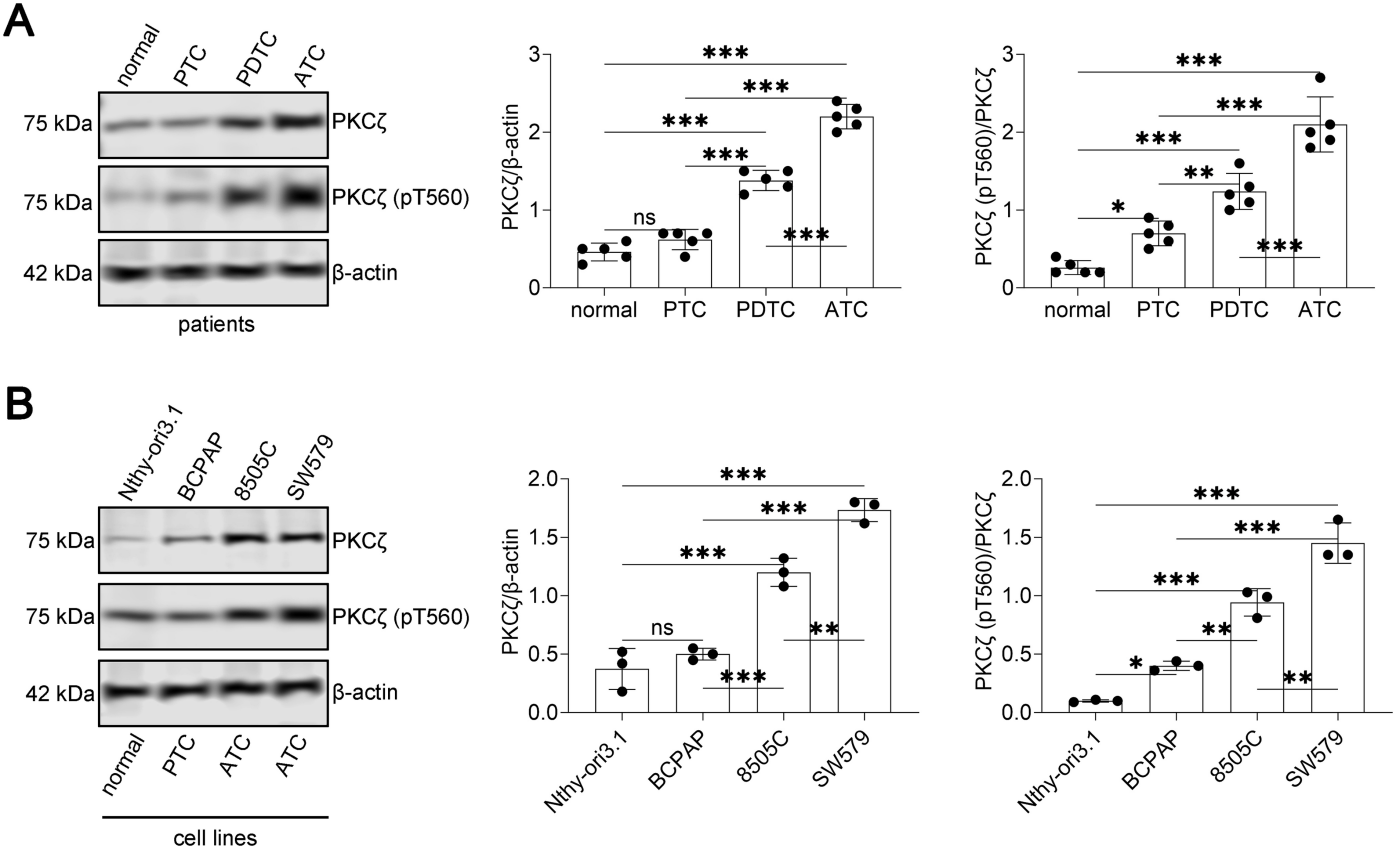
PKCζ expression and phosphorylation levels in thyroid cancer patients and cell lines.

To investigate the role of PKCζ in thyroid cancer progression, we first examined its expression and phosphorylation status in thyroid tissue samples and cell lines. Western blot analysis revealed that both total PKCζ and phospho-PKCζ (pT560) levels were significantly increased in thyroid cancer tissues compared to normal thyroid tissues, with the highest expression observed in ATC, followed by PDTC and PTC (Figure 1A). The ratio of phospho-PKCζ to total PKCζ also showed progressive elevation with increasing tumor aggressiveness. Consistent with these findings, thyroid cancer cell lines exhibited markedly higher PKCζ expression and phosphorylation compared to the normal thyroid cell line Nthy-ori3-1, with 8505C cells showing prominent increase (Figure 1B). These results suggest that PKCζ activation correlates with thyroid cancer malignancy.

(A) Western blot analysis of PKCζ and phospho-PKCζ (pT560) expression in thyroid tissue samples from patients (*n* = 5 per group). Left panel: Representative Western blot images showing PKCζ, phospho-PKCζ (pT560), and *β*-actin (loading control) in normal thyroid tissue, papillary thyroid carcinoma (PTC), poorly differentiated thyroid carcinoma (PDTC), and anaplastic thyroid carcinoma (ATC). Middle panel: Quantification of PKCζ protein expression normalized to β-actin. Right panel: Quantification of PKCζ phosphorylation level expressed as the ratio of phospho-PKCζ (pT560) to total PKCζ. Each dot represents an individual patient sample (complete Western blot data for all 20 patient samples are presented in Supplementary Figure 1).

(B) Western blot analysis of PKCζ and phospho-PKCζ (pT560) expression in thyroid cell lines (*n* = 3 independent experiments). Left panel: Representative Western blot images showing PKCζ, phospho-PKCζ (pT560), and β-actin in normal thyroid cell line (Nthy-ori3-1) and thyroid cancer cell lines (8505C, 8305C, and SW579). Middle panel: Quantification of PKCζ protein expression normalized to β-actin. Right panel: Quantification of PKCζ phosphorylation level expressed as the ratio of phospho-PKCζ (pT560) to total PKCζ. Each dot represents an independent experiment.

Data are presented as mean ± SEM. Statistical significance was determined by one-way ANOVA followed by Tukey’s post-hoc test. Horizontal lines connecting two groups indicate statistical comparisons between those groups. *p < 0.05, **p < 0.01, ***p < 0.001; ns, not significant.

Western blot analysis showing PKCζ, phospho-PKCζ (pT560), and *β*-actin (loading control) expression in thyroid tissue samples from all 20 patients included in this study. Samples are grouped by pathological diagnosis: normal thyroid tissue (*n* = 5), papillary thyroid carcinoma (PTC, *n* = 5), poorly differentiated thyroid carcinoma (PDTC, *n* = 5), and anaplastic thyroid carcinoma (ATC, *n* = 5). Each lane represents an individual patient sample, numbered sequentially within each group. Representative images from this complete dataset are shown in Figure 1A of the main text.

#### ACPD treatment suppresses malignant phenotypes of PDTC cells

Based on our observation that PKCζ expression increases with thyroid cancer progression, we selected two representative cell lines for functional studies: BCPAP (derived from PTC, the most common thyroid cancer subtype accounting for ~80% of cases) and 8505C (derived from PDTC, representing a more aggressive and dedifferentiated phenotype). These cell lines exhibited differential PKCζ expression levels, with 8505C showing higher PKCζ activity than BCPAP, making them ideal models to study both loss-of-function and gain-of-function effects of PKCζ.

**Figure 2:**
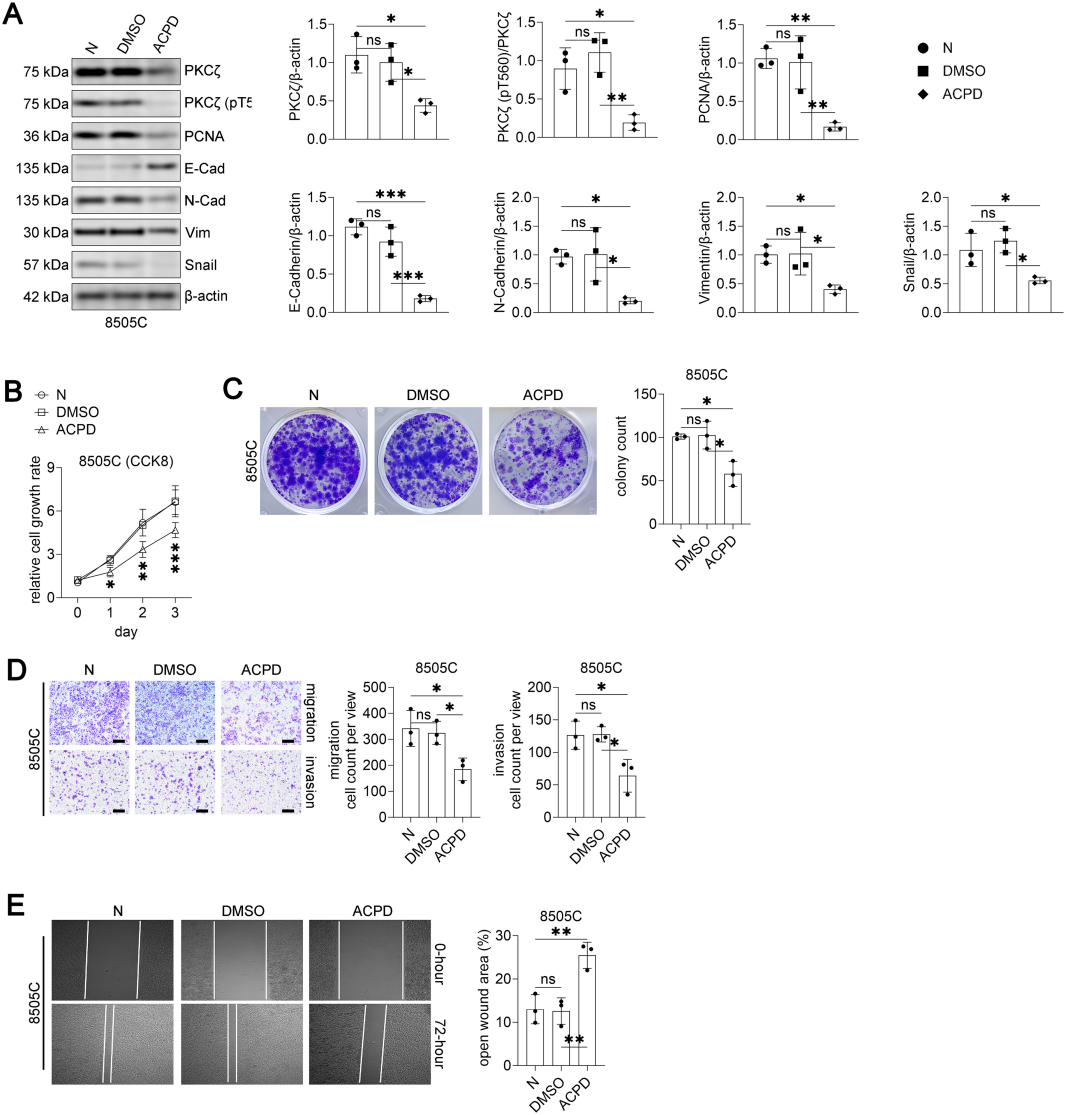
PKCζ inhibitor ACPD suppresses malignant phenotypes in 8505C thyroid cancer cells.

We first investigated whether pharmacological inhibition of PKCζ using ACPD could suppress cancer cell malignancy in the more aggressive 8505C cells. Treatment with ACPD (5 μM) significantly reduced both total PKCζ and phospho-PKCζ (pT560) levels (Figure 2A). ACPD treatment also decreased the expression of proliferation marker PCNA and mesenchymal markers (N-Cadherin, Vimentin, and Snail), while increasing epithelial marker E-Cadherin expression.

Functionally, ACPD treatment significantly inhibited 8505C cell proliferation as assessed by CCK8 assay over a 3-day period (Figure 2B). Colony formation capacity was markedly reduced in ACPD-treated cells compared to vehicle and DMSO controls (Figure 2C). Moreover, ACPD treatment substantially impaired both migration and invasion abilities of 8505C cells in transwell assays (Figure 2D) and decreased wound healing capacity (Figure 2E). These data demonstrate that PKCζ inhibition effectively suppresses multiple malignant phenotypes of PDTC cells.

- Western blot analysis of PKCζ signaling and EMT markers in 8505C cells treated with vehicle control (N), DMSO, or ACPD (2-acetyl-1,3-cyclopentanedione, a specific inhibitor of atypical PKC isoforms) (*n* = 3 independent experiments). Left panel: Representative Western blot images showing PKCζ, phospho-PKCζ (pT560), PCNA, E-Cadherin, N-Cadherin, Vimentin, Snail, and *β*-actin. Right panels: Quantification of protein expression levels normalized to β-actin. Each dot represents an independent experiment.
- Cell proliferation assay using CCK8 method (*n* = 3 independent experiments). 8505C cells were treated with vehicle control (N), DMSO, or ACPD and cell growth was monitored for 3 days. Data are presented as relative cell growth rate compared to day 0. *p < 0.05, **p < 0.01, ***p < 0.001 for ACPD group compared to both N and DMSO groups.
- Colony formation assay. Left panel: Representative images of crystal violet-stained colonies after 14 days of treatment with vehicle control (N), DMSO, or ACPD. Right panel: Quantification of colony numbers (*n* = 3 independent experiments).
- Transwell migration and invasion assays. Left panels: Representative images of crystal violet-stained cells that migrated through uncoated membranes (migration) or invaded through Matrigel-coated membranes (invasion). Scale bars: 100 μm. Right panels: Quantification of migrated and invaded cell numbers per field (*n* = 3 independent experiments).
- Wound healing assay. Left panels: Representative phase-contrast images showing wound closure at 0 and 72 h after scratching. Right panel: Quantification of open wound area at 72 h expressed as percentage of initial wound area (*n* = 3 independent experiments).

All data are presented as mean ± SEM. For panels A, C, D, and E, statistical significance was determined by one-way ANOVA followed by Tukey’s post-hoc test. Horizontal lines connecting two groups indicate statistical comparisons between those groups. *p < 0.05, **p < 0.01, ***p < 0.001; ns, not significant.

#### PKCζ knockdown mimics the anti-tumor effects of ACPD

**Figure 3:**
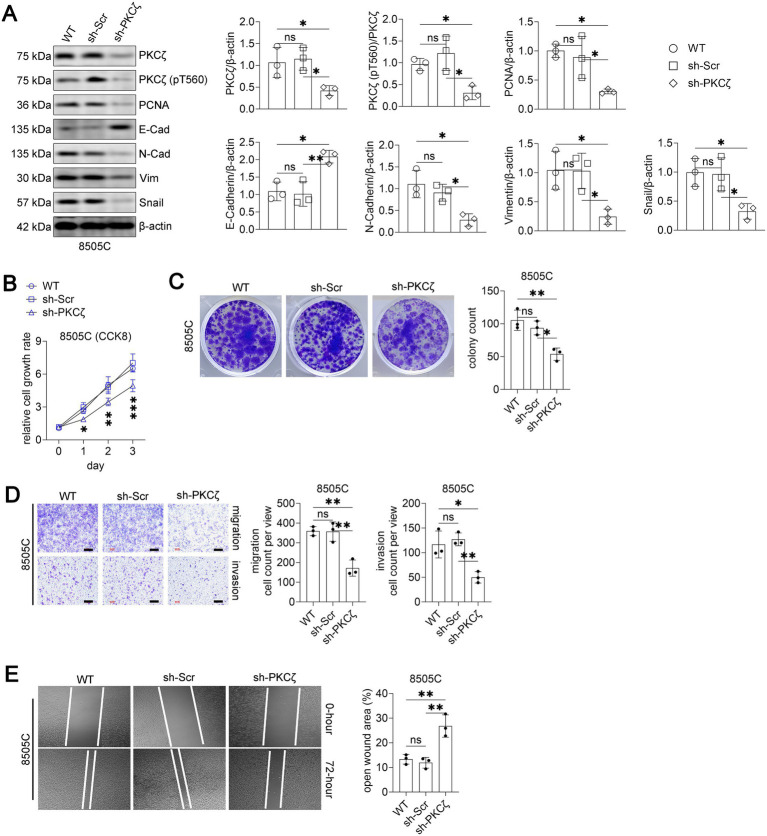
PKCζ knockdown suppresses malignant phenotypes in 8505C thyroid cancer cells.

To validate the specificity of ACPD’s effects through PKCζ inhibition, we generated 8505C cells with stable PKCζ knockdown using shRNA. Western blot analysis confirmed successful knockdown of PKCζ and reduced phospho-PKCζ levels in sh-PKCζ cells compared to wild-type and scrambled control cells (Figure 3A). Similar to ACPD treatment, PKCζ knockdown decreased PCNA, N-Cadherin, Vimentin, and Snail expression while increasing E-Cadherin levels.

PKCζ knockdown significantly suppressed cell proliferation (Figure 3B), colony formation (Figure 3C), migration and invasion (Figure 3D), and wound healing (Figure 3E) compared to control groups. The phenotypic changes induced by PKCζ knockdown closely resembled those observed with ACPD treatment, confirming that ACPD exerts its anti-tumor effects primarily through PKCζ inhibition.

- Western blot analysis of PKCζ signaling and EMT markers in 8505C cells transfected with wild-type (WT), scrambled shRNA (sh-Scr), or PKCζ-specific shRNA (sh-PKCζ) (n = 3 independent experiments). Left panel: Representative Western blot images showing PKCζ, phospho-PKCζ (pT560), PCNA, E-Cadherin, N-Cadherin, Vimentin, Snail, and β-actin. Right panels: Quantification of protein expression levels normalized to β-actin. Each dot represents an independent experiment.
- Cell proliferation assay using CCK8 method (n = 3 independent experiments). 8505C cells transfected with WT, sh-Scr, or sh-PKCζ were monitored for cell growth over 3 days. Data are presented as relative cell growth rate compared to day 0. *p < 0.05, **p < 0.01, ***p < 0.001 for sh-PKCζ group compared to both WT and sh-Scr groups.
- Colony formation assay. Left panel: Representative images of crystal violet-stained colonies after 14 days of culture. Right panel: Quantification of colony numbers (n = 3 independent experiments).
- Transwell migration and invasion assays. Left panels: Representative images of crystal violet-stained cells that migrated through uncoated membranes (migration) or invaded through Matrigel-coated membranes (invasion). Scale bars: 100 μm. Right panels: Quantification of migrated and invaded cell numbers per field (n = 3 independent experiments).
- Wound healing assay. Left panels: Representative phase-contrast images showing wound closure at 0 and 72 h after scratching. Right panel: Quantification of open wound area at 72 h expressed as percentage of initial wound area (n = 3 independent experiments).

All data are presented as mean ± SEM. For panels A, C, D, and E, statistical significance was determined by one-way ANOVA followed by Tukey’s post-hoc test. Horizontal lines connecting two groups indicate statistical comparisons between those groups. *p < 0.05, **p < 0.01; ns, not significant.

#### PKCζ overexpression promotes malignancy in PTC cells and is reversed by ACPD

**Figure 4:**
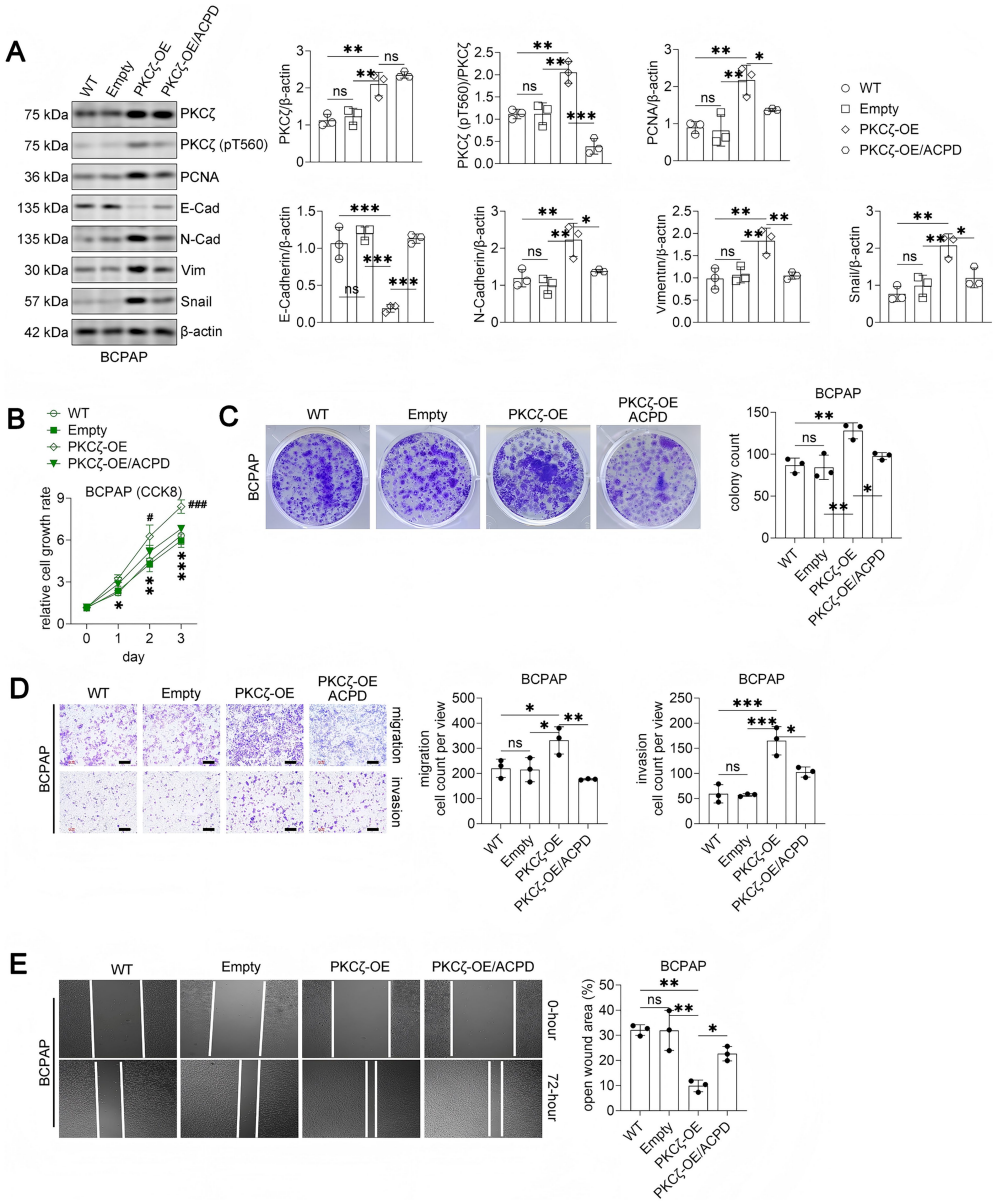
PKCζ overexpression promotes malignant phenotypes in BCPAP cells, which is reversed by ACPD treatment.

To further establish the oncogenic role of PKCζ in thyroid cancer, we overexpressed PKCζ in BCPAP cells, which represent the most common thyroid cancer subtype but have relatively lower endogenous PKCζ levels compared to PDTC cells. PKCζ overexpression significantly increased both total and phosphorylated PKCζ levels, enhanced PCNA and mesenchymal marker expression, and reduced E-Cadherin expression (Figure 4A). These molecular changes were accompanied by increased cell proliferation (Figure 4B), colony formation (Figure 4C), migration and invasion (Figure 4D), and wound healing capacity (Figure 4E).

Importantly, co-treatment with ACPD effectively reversed the pro-tumorigenic effects of PKCζ overexpression, reducing phospho-PKCζ levels and restoring the expression patterns of EMT markers and PCNA to near-control levels. ACPD treatment also significantly attenuated the enhanced proliferation, colony formation, migration, invasion, and wound healing induced by PKCζ overexpression. These findings demonstrate that PKCζ activation is sufficient to promote thyroid cancer cell malignancy and that ACPD can effectively counteract these effects even in PTC cells with forced PKCζ expression.

- Western blot analysis of PKCζ signaling and EMT markers in BCPAP cells transfected with wild-type (WT), empty vector (Empty), PKCζ overexpression vector (PKCζ-OE), or PKCζ-OE combined with ACPD treatment (PKCζ-OE/ACPD) (*n* = 3 independent experiments). Left panel: Representative Western blot images showing PKCζ, phospho-PKCζ (pT560), PCNA, E-Cadherin, N-Cadherin, Vimentin, Snail, and *β*-actin. Right panels: Quantification of protein expression levels normalized to β-actin. Each dot represents an independent experiment.
- Cell proliferation assay using CCK8 method (*n* = 3 independent experiments). BCPAP cells with different treatments were monitored for cell growth over 3 days. Data are presented as relative cell growth rate compared to day 0. *p < 0.05, **p < 0.01, ***p < 0.001 for PKCζ-OE group compared to both WT and Empty groups; #*p* < 0.05, ##*p* < 0.01, ###*p* < 0.001 for PKCζ-OE group compared to PKCζ-OE/ACPD group.
- Colony formation assay. Left panel: Representative images of crystal violet-stained colonies after 14 days of culture. Right panel: Quantification of colony numbers (*n* = 3 independent experiments).
- Transwell migration and invasion assays. Left panels: Representative images of crystal violet-stained cells that migrated through uncoated membranes (migration) or invaded through Matrigel-coated membranes (invasion). Scale bars: 100 μm. Right panels: Quantification of migrated and invaded cell numbers per field (*n* = 3 independent experiments).
- Wound healing assay. Left panels: Representative phase-contrast images showing wound closure at 0 and 72 h after scratching. Right panel: Quantification of open wound area at 72 h expressed as percentage of initial wound area (*n* = 3 independent experiments).

All data are presented as mean ± SEM. For panels A, C, D, and E, statistical significance was determined by one-way ANOVA followed by Tukey’s post-hoc test. Horizontal lines connecting two groups indicate statistical comparisons between those groups. *p < 0.05, **p < 0.01, ***p < 0.001; ns, not significant.

#### PKCζ modulation affects thyroid cancer growth *in vivo*

**Figure 5:**
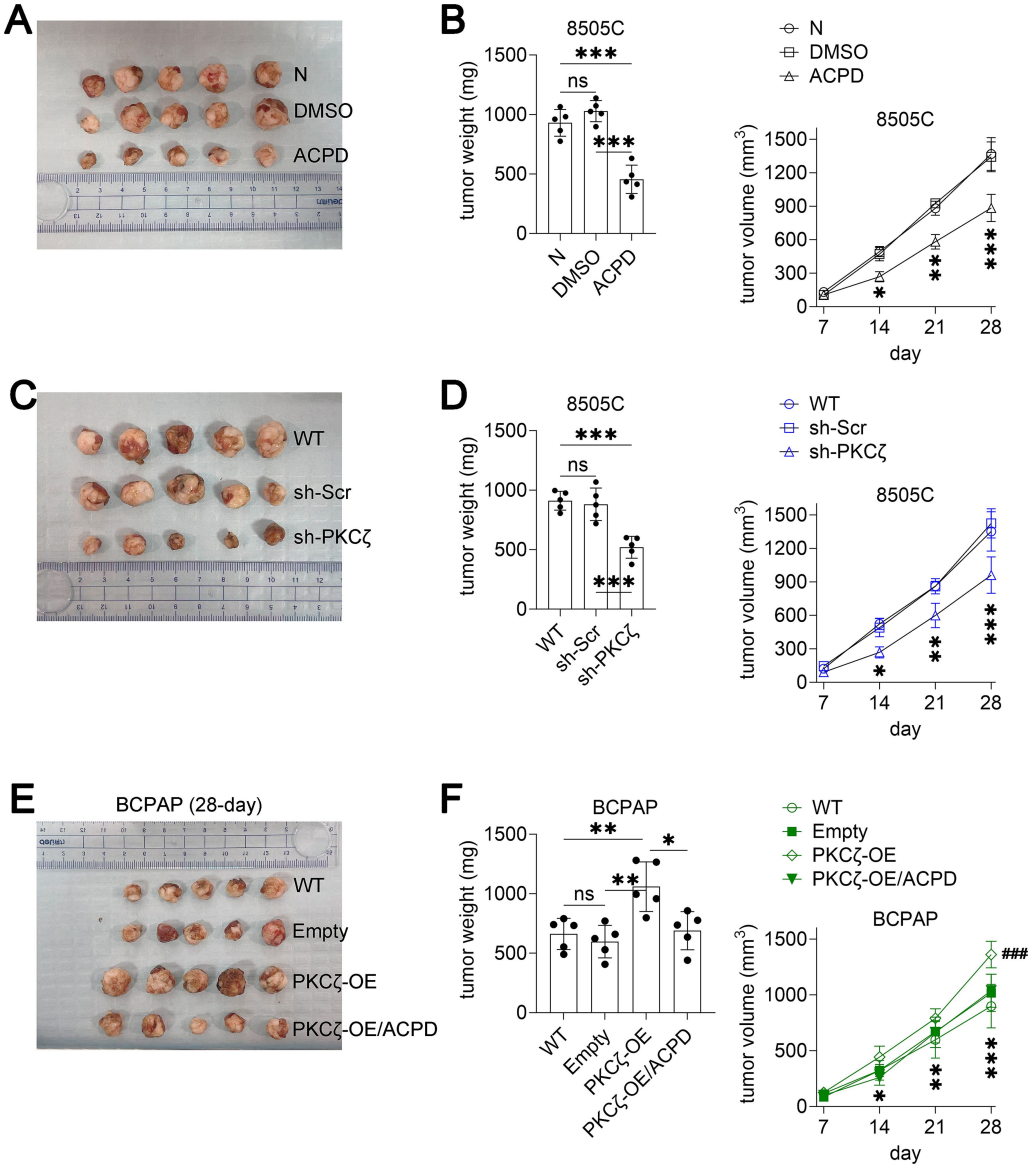
PKCζ inhibition suppresses thyroid cancer growth in xenograft mouse models.

To evaluate the therapeutic potential of PKCζ targeting in vivo, we established xenograft models using both 8505C (PDTC) and BCPAP (PTC) cells. In the 8505C xenograft model, daily treatment with ACPD (10 mg/kg/day) significantly reduced tumor growth and final tumor weight compared to vehicle and DMSO control groups (Figures 5A,B). Similarly, xenografts derived from sh-PKCζ 8505C cells showed markedly reduced tumor growth and weight compared to wild-type and scrambled control groups (Figures 5C,D).

In the BCPAP xenograft model, PKCζ overexpression significantly accelerated tumor growth compared to wild-type and empty vector controls (Figures 5E,F). Notably, ACPD treatment effectively suppressed the enhanced tumor growth induced by PKCζ overexpression, reducing both tumor volume and weight to levels comparable to control groups. These in vivo results corroborate our *in vitro* findings and demonstrate the therapeutic potential of targeting PKCζ in both PTC and PDTC, the two most clinically relevant thyroid cancer subtypes.

(A–B) Effect of ACPD treatment on 8505C xenograft tumor growth (*n* = 5 mice per group). (A) Representative images of excised tumors at the experimental endpoint. (B) Left panel: Tumor weight at the experimental endpoint. Right panel: Tumor growth curves showing tumor volume measured at indicated time points. *p < 0.05, **p < 0.01, ***p < 0.001 for ACPD group compared to both N and DMSO groups.
(C–D) Effect of PKCζ knockdown on 8505C xenograft tumor growth (*n* = 5 mice per group). (C) Representative images of excised tumors at the experimental endpoint. (D) Left panel: Tumor weight at the experimental endpoint. Right panel: Tumor growth curves showing tumor volume measured at indicated time points. *p < 0.05, **p < 0.01, ***p < 0.001 for sh-PKCζ group compared to both WT and sh-Scr groups.
(E–F) Effect of PKCζ overexpression and ACPD treatment on BCPAP xenograft tumor growth (*n* = 5 mice per group). (E) Representative images of excised tumors at day 28. (F) Left panel: Tumor weight at day 28. Right panel: Tumor growth curves showing tumor volume measured at indicated time points. *p < 0.05, **p < 0.01, ***p < 0.001 for PKCζ-OE group compared to both WT and Empty groups; ###*p* < 0.001 for PKCζ-OE/ACPD group compared to PKCζ-OE group.

All data are presented as mean ± SEM. Statistical significance for tumor weight was determined by one-way ANOVA followed by Tukey’s post-hoc test. Tumor growth curves were analyzed using two-way repeated measures ANOVA. Horizontal lines connecting two groups indicate statistical comparisons between those groups. ns, not significant.

## Discussion

The present study provides compelling evidence for the critical role of PKCζ in thyroid cancer progression and demonstrates the therapeutic potential of targeting this kinase. Our findings reveal a progressive elevation of PKCζ expression and phosphorylation from normal thyroid tissue through PTC and PDTC to ATC, suggesting that PKCζ activation correlates with increasing tumor aggressiveness. Importantly, we demonstrate that ACPD, a specific inhibitor of atypical PKC isoforms, effectively suppresses malignant phenotypes in thyroid cancer cells both *in vitro* and *in vivo*. These observations not only establish PKCζ as a key driver of thyroid cancer progression but also provide a strong rationale for developing PKCζ-targeted therapeutic strategies for this disease.

The progression of thyroid cancer from well-differentiated to aggressive forms represents a critical clinical challenge, as advanced tumors are often treatment-resistant with poor prognosis (25, 26). Our data showing progressively increased PKCζ activation from PTC to PDTC and ATC suggest that PKCζ may serve as both a marker and driver of thyroid cancer aggressiveness. The substantially higher PKCζ expression and phosphorylation observed in the PDTC-derived 8505C cell line compared to the PTC-derived BCPAP cells further supports this notion. Notably, pharmacological inhibition or genetic knockdown of PKCζ in PDTC cells resulted in suppression of their aggressive phenotype, including reduced proliferation, migration, and invasion capabilities. These findings suggest that PKCζ activation actively contributes to the maintenance of malignant behaviors in thyroid cancer cells, making it an attractive therapeutic target for advanced thyroid cancers.

A particularly significant finding of our study is the demonstration that PKCζ regulates the epithelial-mesenchymal transition process in thyroid cancer cells. We observed that PKCζ activation promotes the expression of mesenchymal markers including N-Cadherin, Vimentin, and the transcription factor Snail, while suppressing the epithelial marker E-Cadherin. This EMT signature is consistently reversed by either pharmacological inhibition with ACPD or genetic knockdown of PKCζ, underscoring the central role of PKCζ in EMT regulation. The EMT process is fundamental to cancer metastasis and therapeutic resistance, and our findings suggest that PKCζ-mediated EMT may contribute significantly to the aggressive behavior of PDTC and ATC. The ability to reverse EMT through PKCζ inhibition offers a promising strategy to reduce metastatic potential and potentially restore therapeutic sensitivity in advanced thyroid cancers.

ACPD emerges from our study as a promising therapeutic agent with dual efficacy against different thyroid cancer subtypes. The compound demonstrated robust anti-tumor activity not only in PDTC cells with high endogenous PKCζ expression but also effectively counteracted the malignant transformation induced by PKCζ overexpression in PTC cells. This broad spectrum of activity suggests that ACPD could be beneficial across the spectrum of thyroid cancer differentiation states. The *in vivo* efficacy of ACPD in xenograft models provides crucial proof-of-concept for its therapeutic potential. Daily administration of ACPD significantly reduced tumor growth without apparent toxicity, supporting its favorable therapeutic index. Furthermore, the comparable anti-tumor effects observed between ACPD treatment and genetic PKCζ knockdown validate the specificity of ACPD’s mechanism of action and strengthen the rationale for PKCζ-targeted therapy.

From a clinical perspective, our findings have several important implications for thyroid cancer management. The progressive increase in PKCζ activation across different thyroid cancer subtypes suggests its potential utility as a biomarker for risk stratification and therapeutic decision-making. Patients with high PKCζ expression or phosphorylation levels might be candidates for more aggressive treatment approaches or could benefit from PKCζ-targeted therapy. This is particularly relevant for PDTC and ATC patients, who currently have limited therapeutic options and poor prognosis (27, 28). The demonstration that PKCζ inhibition can suppress EMT-related phenotypes is especially significant, as EMT is associated with resistance to radioiodine therapy, a cornerstone of thyroid cancer treatment (29, 30). Therefore, combining PKCζ inhibitors with conventional therapies might overcome treatment resistance and improve outcomes in advanced thyroid cancer.

While our study provides strong evidence for the oncogenic role of PKCζ in thyroid cancer, several limitations should be acknowledged and addressed in future investigations. The precise molecular mechanisms through which PKCζ regulates EMT and other malignant phenotypes require further elucidation, including identification of direct downstream targets and interaction partners. Although ACPD showed promising results in our xenograft models, its efficacy in more complex tumor microenvironments and potential interactions with immune components remain to be explored. Additionally, the development of more selective PKCζ inhibitors with improved pharmacokinetic properties could enhance therapeutic efficacy while minimizing potential off-target effects. Future studies should also investigate the potential of combining PKCζ inhibition with existing therapies, including radioiodine treatment, targeted kinase inhibitors, and immunotherapy, to develop optimal treatment strategies for different thyroid cancer subtypes. Long-term safety studies and evaluation in patient-derived xenograft models or genetically engineered mouse models that better recapitulate human disease progression will be essential steps toward clinical translation. Additionally, although our study demonstrated changes in EMT markers associated with PKCζ modulation, we did not perform direct morphological assessment of cellular differentiation status, such as cell–cell adhesion analysis, aspect ratio measurement, or histopathological evaluation. Future studies incorporating these morphological analyses would provide more comprehensive evidence regarding the role of PKCζ in thyroid cancer differentiation.

In conclusion, our study establishes PKCζ as a critical regulator of thyroid cancer progression and validates its potential as a therapeutic target. The ability of PKCζ inhibition to suppress multiple malignant phenotypes, including EMT, proliferation, and invasion, across different thyroid cancer subtypes highlights its promise for clinical development. These findings provide a foundation for future efforts to develop PKCζ-targeted therapies that could address the urgent clinical need for effective treatments for advanced thyroid cancer.

## Data Availability

The raw data supporting the conclusions of this article will be made available by the authors, without undue reservation.
