# Supplementary figures and images for: Protein kinase C zeta promotes thyroid Cancer progression and represents a novel therapeutic target: evidence from specific atypical PKC inhibitor 2-acetyl-1,3-cyclopentanedione inhibitor studies
Source: Front Med (Lausanne). 2026 Jan 13;12:1714626. doi: 10.3389/fmed.2025.1714626 (PMC12835205; doi:10.3389/fmed.2025.1714626)

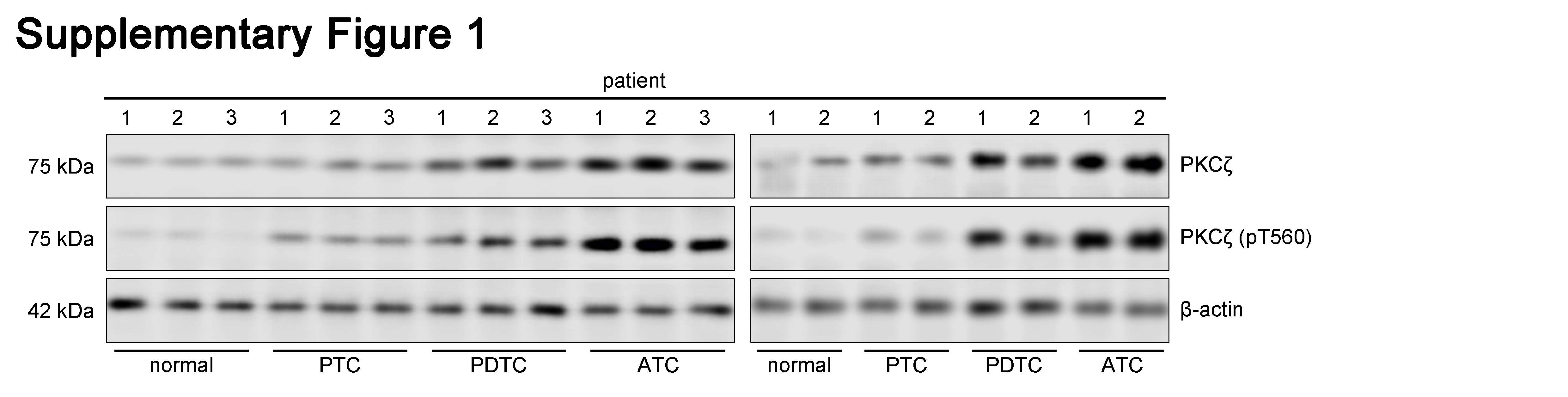

Supplement: Supplementary file 1 [file Image_1.jpg]
